# Inclusion of *Berberis vulgaris* leaf in the diet of fattening lambs: effects on performance, nutrient intake, rumen fermentation, and carcass traits

**DOI:** 10.1093/jas/skad131

**Published:** 2023-04-27

**Authors:** Seyed Morteza Vaghar Seyedin, Navid Ghavipanje, Mohsen Mojtahedi, Seyyed Homayoun Farhangfar, Einar Vargas-Bello-Pérez

**Affiliations:** Department of Animal Science, Faculty of Agriculture, University of Birjand, Birjand 97175-331, Iran; Department of Animal Science, Faculty of Agriculture, University of Birjand, Birjand 97175-331, Iran; Department of Animal Science, Faculty of Agriculture, University of Birjand, Birjand 97175-331, Iran; Department of Animal Science, Faculty of Agriculture, University of Birjand, Birjand 97175-331, Iran; Department of Animal Sciences, School of Agriculture, Policy and Development, University of Reading, Reading RG6 6EU, UK; Facultad de Zootecnia y Ecología, Universidad Autónoma de Chihuahua, 31031 Chihuahua, México

**Keywords:** *Berberis vulgaris*, digestibility, growth performance, in vitro gas production, sustainability

## Abstract

This study was aimed to first, determine the nutritional value of *Berberis vulgaris* leaf (**BVL**), using in vitro gas production technique and second, determine the effect of replacing alfalfa hay (**AH**) with BVL in lamb diets on nutrient intake, performance, and carcass traits. In vitro rumen gas kinetics and fermentation profile were assessed using three fistulated lambs and 96 h incubation of samples. For the in vivo trial, 21 Baluchi male lambs of 5–6 mo of age and 30.6 ± 1.28 kg body weight (**BW**) were randomly assigned to three treatment diets containing BVL at 0% (CTRL), 7.5% (BVL7.5), and 15% (BVL15) of the total dry matter (**DM**) inclusion. The study lasted 84 d, which included 14 d for adaption and 70 d for sample collection. In vitro results showed that BVL had lower gas yield (GY24, *P* ≤ 0.05) than AH. In vivo trial revealed that DM intake increased with BVL15 followed by BVL7.5 (*P* ≤ 0.05). Digestibility of DM, organic matter, NDF, and acid detergent lignin decreased (*P* ≤ 0.05) with BVL15. Total weight gain, average daily gain, hot carcass weight, and cold carcass weight were decreased (*P* ≤ 0.05) with BVL15, however, no differences were observed between the BVL7.5 and CTRL groups. Feed cost per kilogram BW gain decrease (*P* ≤ 0.05) by increasing the inclusion levels of BVL. In conclusion, BVL (up to 7.5% DM of diet) have positive consequences on performance, nutrient intake, rumen fermentation without deleterious effects on fattening performance.

## Teaser

Replacement of alfalfa hay with *Berberis vulgaris* leaf (**BVL**) changed the fermentation parameters in vitro due to its rich bioactive compounds including alkaloids, flavonoids, and tannin. Moreover, the dietary inclusion of BVL (up to 7.5% dry matter of diet) for fattening lambs improved nutrients digestibility and reduced production costs without severe deleterious effects on performance.

## Introduction

The term “Livestock Revolution” was coined to describe the projected increase in demand for animal products due to population growth, rising incomes, and urbanization that will further generate food security challenges, especially in developing countries (Van der Poel et al., 2020; Moorby and Fraser, 2021). Today, there is a huge demand for the use of agro-industrial by-products and residues as a part of livestock diets not only for their relative low cost compared to traditional and expensive feedstuffs but also for higher customer’s demand for safe and healthy animal foods with natural origin (Moorby and Fraser, 2021). Agro-industrial by-products contain great amounts of bioactive compounds, including polyphenols, tannins, alkaloids, and flavonoids that act as growth-stimulants, immune-modulants, and antioxidants (Correddu et al., 2020). Generally, these potential feed staffs are used in livestock diets for cost-effective production that is mainly accompanied by improved performance, animal health, and quality of products, due to their bioactive profile (Correddu et al., 2020; Van der Poel et al., 2020). However, proper testing of options, including a combination of laboratory analysis, controlled-research, and on-farm investigation may be needed to enable farmers for at herd-usage of these feeds (Ghavipanje et al., 2016).

*Berberis vulgaris* is a shrub in the family *Berberidaceae*, native to western Asia, southern and central Europe, and northwest Africa (Salem et al., 2021). Iran with over 11,000 hectares of land under cultivation that produce more than 9,200 tons of dried fruit per year is the world’s largest producer of *B. vulgaris* fruits (Rahimi-Madiseh et al., 2017). *Berberis vulgaris* has an ethnopharmacologically rich history for the treatment of many diseases (Imenshahidi and Hosseinzadeh, 2016; Salem et al., 2021; Ghavipanje et al., 2022). In confirmation of historical pieces of evidence, various in vivo and in vitro studies have shown the anticancer, antimicrobial, antipyretic, antidepressant, antidiabetic, and antioxidant potentials of *B. vulgaris* (Salem et al., 2021; Ghavipanje et al., 2022). Phytochemical analyses have revealed its bioactive constituents with preferably significant amounts of isoquinoline alkaloids (berberine, palmatine, and jatrorrhizine) and bisbenzylisoquinoline alkaloids (Imenshahidi and Hosseinzadeh, 2016; Rahimi-Madiseh et al., 2017; Salem et al., 2021).

The current practice of disposing *Berberis vulgaris* leaf (**BVL**) in landfills and by burning poses an environmental challenge both in terms of soil, air, and water pollution (Ghavipanje et al., 2016; Moghaddam et al., 2021). In this regard, the potential of BVL as a low-cost feed for replacing conventional forages in ruminant diets has been suggested; however, its nutritional value has received little attention (Ghavipanje et al., 2016). Hence, research concerning the potential of this by-product in ruminant diets would be of great value in the term of converting an inherent environmental pollutant into the high-quality animal products for human consumption (Moorby and Fraser, 2021). Ghavipanje et al. (2016) using concurrently in vitro and in vivo approaches showed that the rumen degradability, post-rumen digestibility, and total tract digestibility of BVL is 65.4%, 12.8%, and 69.9% (dry matter [**DM**] basis), respectively. In addition, the inclusion of 17.5% and 34% BVL (DM basis) to cross-breed dairy goats were associated with higher DM intake (**DMI**), milk fat content, and no negative effect on milk production and body weight gain. Interestingly, BVL enhanced antioxidant status of goats (Ghavipanje et al., 2016). Moghaddam et al. (2021) reported that replacing 15% of alfalfa hay (**AH**) with barberry pomace (another by-product of *B. vulgaris*) had no adverse effect on DMI, growth performance, and carcass characteristics of finishing lambs. Moreover, a recent paper by Ghavipanje et al. (2022) reviewed the favorable impact of berberine, the main bioactive compound of BVL, on metabolic impairment of dairy animals, lipid metabolism disorders, oxidative and inflammatory stress, as well as its role as growth and health stimulant.

Despite the promising prospects of agro-industrial by-products usage in ruminant feeding and its effects on boosting food security, in the terms of mitigation food–feed–fuel competition as well as reducing feeding costs (Moorby and Fraser, 2021), there are no many studies, to the best of our knowledge, regarding the use of BVL in ruminant nutrition, the aim of the current study was first, to evaluate in vitro nutritional value of BVL and second, to explore the effects of BVL inclusion in finishing lambs diet, in partial replacement of AH, on performance, carcass characteristics, and ruminal fermentation. We hypothesized that supplementation of ­fattening lambs’ diet with BVL would be a useful strategy to improve growth performance with positive consequences on feeding costs and profitability.

## Materials and Methods

### Ethical considerations and study site

The study was carried out at the experimental farm of the Faculty of Agriculture, University of Birjand, Iran. This farm is located at 1,491 m above sea level with the longitude and latitude, 37.42°N and 57.31°E. The predominant climate in this region is cold semi-arid with a mean annual precipitation and temperature of 171 mm and 18 °C, respectively. All experimental procedures and animal care were in accordance with guidelines of the Iranian Council of Animal Care on the protection of animals used for scientific purposes (protocol ID 19,293).

### Forage collection and preparation

BVL was prepared manually from plant of variety *Berberis vulgaris khorasanica* according to the dominant traditional harvesting method (cutting fruiting shoots) after fruit maturation from gardens around Birjand, South Khorasan, Iran, in November 2020, was sun-dried (35 °C and 0% humidity) to a constant weight. AH was also harvested at the bud stage, sun-dried in the field and stored pending milling and incorporation in lamb diets. Both BVL and AH was shredded using (1 mm) a threshing machine and incorporated into lamb diets.

### In vitro trial

#### Chemical analysis

The predried samples of ingredients, diets, refusals, and feces were ground in a Willey Mill (Arthur Hill Thomas Co., Philadelphia, PA). with a 1-mm mesh sieve then analyzed according to (AOAC, 2015) for DM (method no. 934.01), crude protein (**CP**; method no. 981.10), ash (method 94205), and ether extract (**EE**; method 920.29). The content of neutral detergent fiber (**NDF**) and acid detergent fiber (**ADF**) was determined as described by Van Soest et al. (1991), with the modifications proposed in the Ankom device manual (Ankom Technology Corporation, Macedon, NY). Nonfibrous carbohydrates (**NFC**) were determined with the following equation: NFC = 100 − (CP + NDF + Ash + EE) (NRC, 2007).

The concentrations of total polyphenolic compounds and total tannins (**TT**) in the samples were determined using the Folin–Ciocalteu assay in combination with polyvinyl–polypyrrolidone, using tannicacid (Merck, Damstadt, Germany) as the reference standard (Makkar, 2003). All measurements were done in triplicate.

#### Gas production

Three Baluchi lambs [34.5 ± 2.0 body weight (BW); mean ± SD], fitted with a flexible rumen fistula, fed a total mixed ration (**TMR**) twice daily (0900 and 1600 h), were used as inoculum donors of rumen fluid to carry out in vitro incubation of the feed samples. Ruminal contents of each animal (~200 mL) were obtained immediately before the morning feeding, mixed and strained through four layers of muslin and then kept at 39 °C under a continuous CO_2_ stream. Gas production assay was carried out as described by Menke (1988). Substrates (i.e., BVL and AH) and pure ruminal fluid were used to determine in vitro gas production (Menke, 1988). A total of 0.200 g DM of either BVL or AH was put into glass flasks bottles of 120 mL by triplicate in two tandems and repeated for four incubation runs (total of 24 bottles of each diet), with 30 mL of incubation medium as described by Menke (1988). The volume of gas (mL of gas/g DM) was recorded at 2, 4, 6, 8, 12, 24, 48, 72, and 96 h of incubation and a set of appropriate blanks were included. The pH of the content of each glass flasks was measured using a pH meter (Metrohm 827; pH Lab, Herisau, Switzerland). After incubations, samples were filtered and dried (48 h at 65 °C) to determine the dry matter disappearance. Short chain fatty acid concentrations (**SCFA**) were estimated as follows (Menke, 1988):


$$\begin{array}{l} SCFA\left(mmol\;per\;200\;mg\;DM\right)=0.0222\;GP0.00425 \end{array}$$

where GP is the net gas production in 24 h (mL/200 mg of DM).

### In vivo trial

#### Animals and diets

Twenty-one growing Baluchi male lambs of 5 mo of age with an average initial weight of 30.60 ± 1.28 kg (mean ± SD) were randomly assigned into three diets (seven animals per diet). The lambs were housed in individual (1.50 m × 2.0 m) roofed pens with a raised floor, for 150 d preceded by an adaptation period of 14 d. Prior to the start of the trial, they were weighed and dewormed with vermectin (Silvermec ADE, La Piedad, Mexico), at a dose of 0.1 mL/5 kg live weight, and a vitamin supplement ADE (Vigantol ADE, La Piedad, Mexico) was applied intramuscularly. The diets were formulated by partial and total substitution of AH with BVL and included the following: 1) control, with no BVL (CTRL), 2) BVL7.5, containing 7.5% BVL, and 3) BVL15, containing 15% BVL (DM basis) (Table 1). Diets consisting of 30% forage and 70% concentrate were formulated to meet energy and protein requirements of growing lambs (NRC, 2007), which were isocaloric (contain 2.4 Mcal/kg ME) and isonitrogenous (contain 143 g/kg CP). Throughout the experiment, the TMR diets were offered ad libitum, with feeding levels designed to ensure a daily refusal margin of 10% with free access to the water.

**Table 1.** Ingredient and chemical composition of experimental diets (% DM)

	Experimental diets^1^		
	CTRL	BVL7.5	BVL15
*Ingredients* ^2^			
AH, % DM	30.0	22.5	15.0
BVL^3^, % DM	0.00	7.50	15.0
Corn ground, % DM	16.0	16.0	16.0
Barley ground, % DM	34.0	340.	34.0
Cottonseed meal, % DM	5.00	5.00	5.00
Soybean meal, % DM	3.00	3.00	3.00
Wheat bran, % DM	5.00	5.00	5.00
Beet molasses, % DM	4.00	4.00	4.00
Sodium bicarbonate, % DM	0.50	0.50	0.50
Calcium carbonate, % DM	1.00	1.00	1.00
Salt, % DM	0.50	0.50	0.50
Minerals and vitamins premix^4^, % DM	1.0	1.0	0.0
*Chemical composition of diet*			
Metabolizable energy, Mcal/kg of DM	2.41	2.43	2.44
Ether extract, % DM	2.11	2.18	2.25
Crude protein, % DM	14.3	14.3	14.3
Ash, % DM	6.94	6.89	6.82
NDF, % DM	27.9	27.4	27.0
ADF, % DM	17.1	16.0	14.9
NFC^5^, % DM	48.7	49.2	49.7

^1^Control, BVL7.5, and BVL15 contained 0%, 7.5%, and 15% BVL (DM basis), respectively.

^2^AH, alfalfa hay; BVL, *Berberis vulgaris* leaf; NDF, neutral detergent fiber; ADF, acid detergent fiber; NFC, nonfiber carbohydrates; DM, dry matter.

^3^Contain of 95.5% DM, 13.80% crude protein, 2.13% ether extract, 7.88% Ash, 34.27% NDF, 17.97% ADF (DM basis).

^4^Containing vitamin A (250,000 IU/kg), vitamin D (50,000 IU/kg), and vitamin E (15000 IU/kg), manganese (2.25 g/kg), calcium (120 g/kg), zinc (7.7 g/kg), phosphorus (20 g/kg), magnesium (20.5 g/kg), sodium (18.6 g/kg), iron (1.25 g/kg), sulfur (3 g/kg), copper (1.25 g/kg), cobalt (14 mg/kg), iodine (56 mg/kg), and selenium (10 mg/kg).

^5^Nonfibrous carbohydrates were estimated according to the equation: NFC = 100 – (NDF + CP + EE + Ash).

#### Sampling and measurements

During the experiment, lambs were fed individually twice daily (0700 and 1700 h) and amounts of diet fed and refusals were weighed daily to determine DMI by subtracting the quantity fed minus refusals multiplied by the DM content of the TMR. All lambs were weighed individually before the morning feeding on days 0, 28, 56, and 84 to determine the average daily gain (**ADG**). Feed conversion ratio (**FCR**) and feed efficiency (**FE**) was calculated as follows: FCR = [DMI, (kg/d)/ADG, (kg/d)]; FCR = [ADG, (kg/d)/DMI, (kg/d)]. An estimate was also made of the feeding cost per kilogram of body weight gain for each dietary group.

Acid insoluble ash (**AIA**) content was used as an internal marker to determine the apparent digestibility of DM, organic matter (**OM**), EE, CP, and NDF as reported Van Keulen and Young (1977). Briefly, feed and feces were sampled three times (each in three consecutive days). Then 5 g of feces or feed sample was subjected to a temperature of 550 to 600 °C for 3 h. Then the resulting ash was boiled with 100 mL of 2 N HCl for 5 min. Next, it was filtered with a filter paper and after washing three times with distilled water, it was subjected to a temperature of 550 to 600 °C for another 3 h. The resulting residual was calculated as AIA (Van Keulen and Young, 1977).

Samples of rumen fluid were collected from lambs on days 28, 56, and 84 at 2 h after the morning feeding using a stomach tube attached to an Erlenmeyer flask connected to vacuum pump. The rumen fluid was filtered through four layers of cheesecloth, and pH was determined immediately using a portable pH meter (Metrohm 827; pH Lab, Herisau, Switzerland). Ruminal NH_3_–N concentration was determined according to the procedure by Broderick and Kang (1980).

At the end of 84-d trial period, lambs were weighed after 14 h of feed deprivation and then were slaughtered. Hot carcass weight (**HCW**) was recorded immediately after slaughtering and cold carcass weight (**CCW**) was recorded 24 h after slaughtering. Dressing percentage was calculated using HCW weight. Carcasses were chilled at 4 °C for 24 h and then weighed again, so that chilling losses were calculated as the difference between HCW and CCW expressed as a proportion of HCW. The weights of liver, lung, heart, kidney, and testicles were measured using calibrated scale (ASA2200, Sepahan Towzin Co., Isfahan, Iran). Also, kidney fat visceral fat, kidney fat, and tail fat were weighed. The left side of each carcass was divided into five anatomical regions as described by Colomer-Rocher et al. (1987).

### Statistical analysis

In vitro gas production and fermentation profile data statistically analyzed using the GLM procedure of Statistical Analysis System software version 9.2 (SAS/STAT, SAS Institute Inc., Cary, NC) using following model: *y*_*ij*_ = μ + *T*_*i*_ + *e*_*ij*_, where *y*_*ij*_ is the dependent variable, μ is the overall mean, *T*_*i*_ is the treatment effect, and *e*_*ij*_ is the experimental error.

In vivo data were analyzed based on a completely randomized design with three treatments (diets) and seven replicates (lambs). All data were analyzed by a mixed model for repeated measurements. The statistical model was: *y*_*ijkl*_ = μ + *T*_*i*_  + *A*_*j*(*i*)_ + *W*_*k*_ + *T*_*i*_ × *W*_*k*_ + *e*_*ijkl*_, where *y*_*ijkl*_ is the dependent variable, μ is the overall mean, *T*_*i*_ is the fixed effect of diets, *A*_*j*(*i*)_ is the random effect of *j*th lamb within *i*th diet, *W*_*k*_ is the fixed effect of repeated measurements, *T*_*i*_ × *W*_*k*_ is the interaction fixed effect between *T*_*i*_ and *W*_*k*_, and *e*_*ijkl*_ is the residual error. The covariance structure with the best fit was the unstructured. Least-square means were computed and tested for differences by the Tukey’s test. Differences of least-square means were considered to be significant at *P *≤ 0.05, and that of *P *≤ 0.10 was considered as a tendency.

## Results

### In vitro experiment

#### Chemical composition

BVL had more DM (*P* = 0.0010), EE (*P* = 0.0001), and NFC (*P* = 0.0001), but less CP (*P* = 0.0008), NDF (*P* = 0.0001), ADF (*P* = 0.0001), and ash (*P* = 0.0057) than AH (Table 2). Regarding polyphenol compounds, the contents of phenolic compounds (**PC**) (*P* = 0.0001) and TT (*P* = 0.0001) were higher in BVL than AF.

**Table 2.** Chemical composition of alfalfa hay (AH) and *Berberis vulgaris* leaf (BVL)^1^

Item^2^	Forages		SEM^3^	*P*-value
	AH	BVL		
DM, %	93.53^b^	95.50^a^	0.0362	0.0010
CP, % DM	14.44^a^	13.81^b^	0.0492	0.0008
EE, % DM	1.21^b^	2.13^a^	0.0131	0.0001
Ash, % DM	9.86^a^	7.88^b^	0.0260	0.0057
NDF, % DM	40.72^a^	34.27^b^	0.0404	0.0001
ADF, % DM	32.39^a^	17.97^b^	0.4324	0.0001
NFC, % DM	33.76^b^	41.91^a^	0.2791	0.0001
PC, % DM	12.43^b^	14.59^a^	0.0269	0.0001
TT, % DM	0.74^b^	5.94^a^	0.0174	0.0001

Within row, different letters (a, b) indicate difference between diets (*P* ≤ 0.05).

^1^Values are least-square means.

^2^DM, dry matter; CP, crude protein; EE, ether extract; NDF, natural detergent fiber; ADF, acid detergent fiber; NFC, nonfiber carbohydrates; PC, phenolic compounds; TT, total tannins.

^3^Pooled standard error of the mean.

#### In vitro gas kinetics

Gas production at 48 and 96 h was higher for AH than BVL. Also, the AH presented higher dry matter degraded substrate (DMD96h, *P* = 0.008) and gas yield (GY24, *P* = 0.027) (Table 3). However, there were no differences for production of SCFA (*P* = 0.163) and microbial CP (*P* = 0.136).

**Table 3.** In vitro rumen gas kinetics (mL gas/g DM) and fermentation profile alfalfa hay (AH) and *Berberis vulgaris* leaf (BVL)^1^

Item^2^	Forages		SEM^3^	*P*-value
	AH	BVL		
*In vitro gas kinetics*				
VF_a_	41.130^a^	10.007^b^	1.5221	0.0001
VF_b_	21.444^b^	38.505^a^	1.4675	0.0001
C_a_	0.060^b^	0.082^a^	0.0037	0.0001
C_b_	0.051^a^	0.018^b^	0.0038	0.0001
Lag time	8.972^a^	5.932^b^	0.0478	0.0001
*In vitro gas production*				
12 h	10.379	11.340	0.6742	0.5351
24 h	24.984	22.130	1.4000	0.1635
48 h	49.365^a^	38.418^b^	2.3405	0.0032
96 h	64.134^a^	48.926^b^	2.6106	0.0005
*Fermentation parameters*				
DMD96	728.873^a^	646.485^b^	12.1595	0.0087
GY24	62.716^a^	52.395^b^	2.1488	0.0274
SCFA	0.106	0.094	0.0062	0.1635
MCP	342.261	329.401	4.8830	0.1361
pH24	7.64^a^	7.40^b^	0.0078	0.0001

Within row, different letters (a, b) indicate difference between diets (*P* *≤* 0.05).

^1^Values are least-square means.

^2^a_1_= gas volume derived from the degradation of the rapid digestion soluble fraction when ‘t→∞’, a_2_= gas volume derived from the degradation of the slow digestion insoluble fraction when ‘t→∞’, c_1_= rate of gas production due to the degradation of the soluble fraction (ml/h), c_2_= rate of gas production due to the degradation of the insoluble fraction (ml/h), t= incubation time (h), Lag time = the initial delay before gas production begins (h); DMD96 = DM degraded substrate (mg/g DM); GY24 = gas yield at 24 h (mL gas/g DMD); SCFA = short chain fatty acids (mmol/g DM); MCP = microbial CP production (mg/g DM).

^3^Pooled standard error of the mean.

### In vivo experiment

#### Nutrient intake, digestibility, and growth performance

The highest (*P* = 0.0001) DMIs were observed in lambs fed with BVL15 followed by BVL7.5 (Table 4). Likewise, dietary inclusion of 15% BVL (DM basis, BVL15) increased the intakes of OM (*P* = 0.0001), NDF (*P* = 0.0001), ADF (*P* = 0.0001), and acid detergent lignin (**ADL**; *P* = 0.0001) while no difference was observed between the BVL7.5 and CTRL.

**Table 4.** Intake, nutrient digestibility, growth performance, and feeding cost in fattening lambs fed *Berberis vulgaris* leaf (BVL) included diets^1^

Item^2^	Diets^5^			SEM^6^	*P*-value
	CTRL	BVL7.5	BVL15		
*Intake*					
DM intake, g/d	874.49^b^	896.88^b^	961.50^a^	8.7723	0.0001
OM intake, g/d	817.59^b^	835.09^b^	895.35^a^	8.1604	0.0001
Fat intake, g/d	126.00^b^	128.25^b^	137.44^a^	1.2576	0.0001
NDF intake, g/d	18.62^c^	19.55^b^	21.05^a^	0.1996	0.0001
ADF intake, g/d	244.64^b^	246.01^b^	263.09^a^	2.4982	0.0001
ADL intake, g/d	148.82^ab^	143.59^b^	152.48^a^	1.8829	0.0126
*Digestibility*					
DM, g/kg	70.40^a^	70.62^a^	66.78^b^	0.7247	0.0161
OM, g/kg	72.14^a^	71.71^a^	68.18^b^	0.2167	0.0001
NDF, g/kg	75.92^a^	74.87^a^	71.78^b^	0.3272	0.0003
ADF, g/kg	75.20	74.74	74.63	0.2591	0.3217
ADL, g/kg	55.46^a^	55.25^a^	51.68^b^	0.6544	0.0109
*Growth performance*					
IBW, kg	30.17	30.78	30.24	0.4088	0.5215
FBW, kg	43.40^a^	44.10^a^	40.77^b^	0.3955	0.0001
Total BW gain, kg	13.22^a^	13.31^a^	10.52^b^	0.2436	0.0001
ADG, g/d	176.38^a^	177.52^a^	140.38^b^	3.2480	0.0001
FCR	4.98^b^	5.06^b^	6.85^a^	0.0997	0.0001
FE	0.20^a^	0.19^a^	0.14^b^	0.0034	0.0001
Feed cost per kg BW gain, USD ^3,4^	1.83^b^	1.77^b^	2.26^a^	0.0348	0.0001
Return per kg BW gain, USD ^3,4^	23.85^a^	24.92^a^	14.44^b^	0.8378	0.0001

Within row, different letters (a, b) indicate difference between diets (*P* ≤ 0.05).

^1^Values are least-square means.

^2^DM, dry matter; OM, organic matter; NDF, natural detergent fiber; ADF, acid detergent fiber; ADL, acid detergent lignin; IBW, initial body weight; FBW, final body weight; ADG, average daily gain; FCR, Feed conversion ratio; FE, feed efficiency; BW, body weight.

^3^Each kilogram of AH and BVL was 0.270 and 0.020 USD.

^4^Calculations are made with the following exchange: 1 USD = 48,100 IR Rials.

^5^CTRL, BVL7.5, and BVL15 contained 0%, 7.5%, and 15% BVL (DM basis), respectively.

^6^SEM, Pooled standard error of the mean.

No differences were observed between diets on ADF digestibility (*P* = 0.321, Table 4). Digestibility of DM (*P* = 0.016), OM (*P* = 0.001), NDF (*P* = 0.003), and ADL (*P* = 0.012) decreased with BVL15; however, no differences were observed between the BVL7.5 and CTRL groups.

Regarding the animal performance results, the average initial BW of the lambs from each treatment had no difference (*P* = 0.521, Table 4). Animals fed BVL15 diet showed a lower final BW (**FBW**) compared with BVL7.5 and CTRL groups (*P* = 0.0001). The lambs fed BVL7.5 and BVL15 had approximately 1.61% higher and 6.06% lower FBW compared to the CTRL group. Likewise, total weight gain (*P* = 0.0001) and ADG (*P* = 0.0001) decreased with BVL15; however, no differences were observed between the BVL7.5 and CTRL groups. In addition, higher FCR values were found in BVL15 treatment (*P* = 0.0001). Feed cost per kilogram BW gain decrease (*P* = 0.0001, Table 4) by the increasing inclusion levels of BVL in the experimental diets. In addition, there were no differences between BLV7.5 and CTRL for the return per kilogram BW gain; however, BVL15 was associated with lower return (*P* = 0.0001).

#### Rumen fermentation

No significant effects between diets (*P* > 0.05) were observed regarding pH and rumen ammonia nitrogen (NH_3_) concentration during the three times of sampling (i.e., days 28, 56, and 84 of the trial, Table 5).

**Table 5.** Ruminal fermentation parameters in fattening lambs fed *Berberis vulgaris* leaf (BVL) included diets^1^

Item	Day	Diets^2^			*P*-value	SEM^3^
		BVL15	BVL7.5	CTRL		
pH	28	6.28	6.39	6.53	0.5461	0.1528
	56	6.19	6.26	6.34	0.7881	0.1473
	84	6.10	6.12	6.14	0.9935	0.2074
	Total	6.19	6.26	6.33	0.5776	0.0957
NH_3_	28	14.23	15.53	15.16	0.4443	0.6972
	56	15.11	14.03	14.40	0.5723	0.7012
	84	15.30	14.40	14.03	0.4006	0.6302
	Total	14.88	14.65	14.53	0.8132	0.3871

Within row, different letters (a, b) indicate difference between diets (*P* ≤ 0.05).

^1^Values are least-square means.

^2^CTRL, BVL7.5, and BVL15 contained 0%, 7.5%, and 15% BVL (DM basis), respectively.

^3^Pooled standard error of the mean.

#### Carcass characteristics

The carcass yield and metrics are presented in Table 6. The animals with the highest finishing weight were those of the BVL7.5 group, and the BVL15 group had the lowest weight (*P* = 0.007).

**Table 6.** Carcass characteristics in fattening lambs fed *Berberis vulgaris* leaf (BVL) included diets^1^

Item^2^	Diets^3^			*P*-value	SEM^4^
	BVL15	BVL7.5	CTRL		
FBW, kg	43.40^a^	44.10^a^	40.77^b^	0.3955	0.0070
HCW, kg	21.18^a^	21.83^a^	19.72^b^	0.2984	0.0065
CCW, kg	20.19^a^	20.08^a^	18.93^b^	0.2065	0.0092
Dressing BW, kg	17.5^a^	16.4^a^	15.9^b^	0.281	0.0162
Leg, kg	6.22	6.14	5.96	0.107	0.3054
Flank, kg	2.74	2.75	2.64	0.034	0.1085
Shoulder, kg	0.952	0.946	0.947	0.019	0.9717
Head, kg	2.82	2.86	2.87	0.032	0.5559
Heart, kg	0.233	0.228	0.240	0.004	0.1918
Liver, kg	1.03	1.06	1.08	0.024	0.4181
Kidney, kg	0.341	0.308	0.280	0.016	0.0950
Lungs, kg	0.486	0.499	0.495	0.009	0.6605
Testis, kg	0.182	0.184	0.183	0.003	0.8990
Tail fat, kg	3.68^a^	3.58^a^	2.89^b^	0.127	0.0093
Visceral fat, kg	0.240^a^	0.218^a^	0.158^b^	0.009	0.0023

Within row, different letters (a, b) indicate difference between diets (*P* ≤ 0.05).

^1^Values are least-square means.

^2^FBW, final body weight; HCW, hot carcass weight; CCW, cold carcass weight; BW, body weight.

^3^CTRL, BVL7.5, and BVL15 contained 0%, 7.5%, and 15% BVL (DM basis), respectively.

^4^Pooled standard error of the mean.

The hot carcass weight (*P* = 0.006), cold carcass weight (*P* = 0.009), and dressing BW (*P* = 0.016) were decreased with BVL15. Regarding the effect of diets on carcass cuts, there were no effects on weights of shoulder (*P* = 0.971), leg (*P* = 0.305), flank (*P* = 0.108), and weights of the internal organs [including heart (*P* = 0.191); liver (*P* = 0.418), kidney (*P* = 0.095), lungs (*P* = 0.660), and testis (*P* = 0.899), Table 6]. However, tail fat (*P* = 0.009) and visceral fat (*P* = 0.002) decreased (with 15% BVL inclusion to the diets.

## Discussion

Unfortunately, more than 820 million people suffered from hunger in 2020, and about 2 billion people undergo moderate or severe food insecurity globally (Van der Poel et al., 2020; Salem et al., 2021). With the continuously increasing global population, livestock production has been projected to expand over 21% by 2025 (Van der Poel et al., 2020). Given the challenges facing worldwide livestock production, namely, limited availability of natural resources, ongoing climatic changes, and food–feed–fuel competition, recently, agricultural by-products, resulting from crops, fruit and ­vegetable processing which can be used in animal diets have become a hot topic in the animal feed industry (Moorby and Fraser, 2021). In this sense, in this study, AH was partially replaced by BVL, which is normally less expensive than AH. It is well documented that BVL has a great potential as animal feed (Ghavipanje et al., 2016; Moghaddam et al., 2021; Ghavipanje et al., 2022), but to the best of our knowledge, no investigation has been addressed BVL in fattening lambs. In this study, we found that BLV inclusion (up to 7.5% DM of diet) in fattening lambs improved nutrients digestibility and reduced production costs without severe deleterious effects on performance.

### In vitro experiment

In the present study we investigate the chemical composition and in vitro rumen fermentation profile of BVL which is the first step in determining the usefulness of feed in ruminant production systems. Our results showed that the BVL contained 93.5% DM, 14.4% CP, 40.8% NDF, 32.4% ADF, 33.7% NFC, and 1.21% EE; this finding was similar to that of Ghavipanje et al. (2016), who stated that BVL contained 94.02% DM, 9.98% CP, 29.52% ash, 30.89% NDF, 20.03% ADF, and 2.36% EE. Similarly, the chemical composition of BVL in the present study was consistent with the previous reports (Mokhtarpour et al., 2013). However, it should be noted that BVL shows variations in chemical composition depending on the soil, climatic situation, and stage of maturity (Ghavipanje et al., 2016).

The chemical composition of feed is an essential factor for predicting the true digestibility of dry matter or organic matter in in vitro gas production research (Getachew et al., 1998; Jayanegara et al., 2017). In theory, the greater the DM degradability, the greater the gas production (Getachew et al., 1998). The gas production of fraction b was higher in BVL than AH. Regarding to the degradation rate of DM (fraction *C*_*a*_), BVL presented higher rates than AH, indicating that forage require less time inside the rumen to reach their maximum degradation potential (Amorim et al., 2020). It is well established that the in vitro rumen gas kinetics and fermentation profile of a plant spices is highly related to its chemical composition, tannin content and digestibility (Amorim et al., 2020; dos Santos et al., 2020). Gas in the in vitro system is formed mainly from carbohydrate (Getachew et al., 1998). Higher GP in BVL than AH is closely related to NDF content of BVL, since the GP was positively correlated with plant cell wall (Jayanegara et al., 2017). However, The higher *C*_*b*_ values of AH was possibly due to the lower cell wall components, which has negative influence on DM degradability (dos Santos et al., 2020). It was expected that the volume of gas production in the BVL would be higher than AH due to the higher amount of NFC (41.91 vs. 33.76 g/100 g DM) and the lower amount of NDF (34.27 vs. 40.72 g/100 g DM); however, the contradictory results observed. Probably, higher PC and TT in BVL reduced the degradability potential, since the plant secondary metabolites (**PSMs**) can lead to a significant difference in fermentation and gas production (Getachew et al., 1998). The change in the lag phase is mainly due to the proliferation of the microbial population and their placement on the feed particles to form a biofilm colony (Getachew et al., 1998).

### In vivo experiment

The inclusion of 7.5% and 15% BVL (DM basis) to the diets resulted in 2.56% and 9.95% higher DMI compared to CTRL group, respectively. Likewise, the intakes of OM, NDF, ADF, and ADL were enhanced with inclusion of 15% BVL (DM basis, BVL15). As shown in previous studies, the addition of BVL to the diets enhances DMI and nutrient digestibility in sheep (Moghaddam et al., 2021) and goat (Ghavipanje et al., 2016). In contrast, we found that digestibility of DM, OM, and NDF decreased with BVL15 in the present study. It has been well documented that using agro-industrial by-products with great amounts of PSMs could affect nutrient digestibility (Correddu et al., 2020; Soltan et al., 2021). PSMs, especially tannins, may have beneficial or detrimental effects on nutrient digestion depending on type, quantity consumed, compound structure, molecular weight, and the physiological status of the consuming species (Barry and McNabb, 1999; Benchaar et al., 2008). In confirmation, (Barry and Forss, 1983) reported that tannins (>50 g/kg DM) can adversely affect nutrient digestibility, and this limits the use of alternative feed resource. Decreases of digestibility with tannins have been already reported either in cows (Barry and McNabb, 1999) and sheep (Priolo et al., 2000). Therefore, the depressing effect of dietary inclusion of 15% BVL for fattening lambs in the current study might be due to negative feedback of tannins.

Our results indicated that the inclusion of BVL up to 7.5% DM did not affect FBW, ADG, FCR, and FE. In contrast, lambs fed the BVL up to 15% DM of diet had a reduction in total BW gain (20.4% compared to the CTRL group and 20.9% compared to BVL7.5) resulting to lower ADG and higher FCR. It is well accepted (Nasri et al., 2011) that the reduction in diet digestibility negatively affects weight gain. Although the tannin levels of BVL were low, it had an adverse effect on the growth performance of lambs. In this study, lower ADG in animal fed BVL up to 15% DM of diet is probably attributed to the PSMs (especially tannins) in BVL due to long-term exposure (84 d) that decrease nutrient digestion and further increases FCR. Moreover, total BW gain and ADG in lambs fed BVL up to 7.5% DM were numerically higher and this could provoke different rates of digestion, which might enhance the efficiency of energy utilization and consequently enhance growth rate (Soltan et al., 2021). Analysis of feeding costs showed that inclusion of 7.5% BVL to diet (DM basis) led to a sensible reduction of the feed cost per kilogram BW gain (3.28% reduction compare with CTRL). The concentrations of ammonia-N and ruminal pH of lambs were not changed with dietary inclusion of 7.5% or 15% BVL (DM basis). Not only there is no report concerning the effect of BVL feeding on ruminal fermentation parameters in ruminants, but also previous findings on dietary inclusion of or PSMs on ruminal NH_3_ concentrations and pH has been inconsistent. Similar with our findings, it has been shown that the PSMs had no effects on the ruminal pH and ammonia-N concentrations (Benchaar et al., 2008; Zhou et al., 2012). However, some researchers reported that ruminal ammonia-N concentrations decreased linearly with increasing level of PSMs (Nasri et al., 2011). However, the lack of effect on rumen fermentation parameters following inclusion of BVL to the diet of fattening lambs indicates that this by-product did not interrupt the rumen fermentation function. It has been reported that inhibition of digestive enzymes and prevention of adhesion of rumen microorganisms in the presence of tannins can reduce the degradability of feed (Saminathan et al., 2016). Low amounts of tannin (3% to 4%) have beneficial effects in preventing excessive breakdown of soluble proteins in the rumen and increasing the flow of essential amino acids to the intestine, however, high amounts of tannin (6% to 10%) in laboratory conditions and it has also had negative effects on animals (Aboagye et al., 2018). In several studies, the dietary inclusion of plant-derived antioxidants led to an increase in feed consumption (Emami et al., 2015; Hukerdi et al., 2019). The high content of hydrolyzable tannins in barberry leaves has no effect on feed consumption compared to products such as pistachio (66.8 mg/g DM) or olive by-products (56.85 mg/g DM) (Hukerdi et al., 2019). Also, antimicrobial activity has been reported at low levels when consuming flavonoids (Li et al., 2022). In fact, flavonoids as PSMs can inhibit the activity of Gram-negative and Gram-positive bacteria and even protozoa by disrupting cell integrity (Xie et al., 2015). Probably, the long-term (84 d) exposure of livestock to flavonoids, along with changes in microbial flora, increased feed consumption, weight loss and reduced digestibility. Altogether, our results indicated a favorable effect of BVL inclusion on the DMI of fattening lambs. Moreover, there were no differences between BVL7.5 and CTRL in regard of nutrient digestion, ADG, and FCR that along with lower feed cost per kilogram BW gain make BVL7.5 rationalization in fattening lambs diet.

The yield of most of the edible offal components did not change with dietary inclusion of BVL (at 7.5% and 15%, DM basis) to *Baluchi* lambs. Generally, dressing percentage of lamb ranges between 40% and 50%, which is corroborated by the findings in the current study. Dressing percentage is an important parameter in the assessment of meat-producing animals since it determines the amount of meat available (Moghaddam et al., 2021). HCW and CCW decreased with inclusion of 15% BVL; however, in lambs fed BVL up to 7.5% DM of diet, the HCW were numerically higher than CTRL (with an increase of 5.77%). In addition, there were no dietary differences in terms of head, shoulder, brisket, flank, and leg weights as well as internal organs. This similarity in weights of carcass cuts can be attributed to the similar initial weight, ADG, and slaughter weight among the lambs. Similarly, Moghaddam et al. (2021) reported that inclusion barberry pomace (another by-product of barberry) at 7.5% and 15% of dietary DM did not affect weight of carcass, commercial cuts (neck, shoulder, loin, leg, fat-tail, brisket, flank), and noncarcass components (head, skin, feet, lung and trachea, heart, liver, spleen, gastro-intestinal, kidney, and bladder) but linearly increased HCW and CCW of *Baluchi* lambs. Moreover, consists with our results, pervious findings (Valizadeh et al., 2010; SoltaniNezhad et al., 2016) with feeding agro-industrial by-products did not observe any changes in the carcass characteristics of lambs. Hence, improved HCW without unfavorable effects on carcass in lambs receiving 7.5% BVL-containing diets demonstrated that it could be a useful feeding strategy for mitigating the impact of feed costs, competition with food intended for human consumption, and the environmental impact of waste sources. It is important to note that the BVL depend on local and seasonal availability and may be produced and procured by small or medium-size entrepreneurs, resulting in fluctuating availability. Our results create a benchmark for the use of BVL for fattening performance of lambs at different doses. However, further research should be performed using in vivo studies to analyze rumen function and quality of products.

## Conclusions

The dietary inclusion of BVL at 7.5% and 15% DM of diet enhanced DMI in fattening lambs and affected apparent digestibility. The relationship between nutrient intake and digestibility (DM, OM, NDF, and ADF) was optimal with 7.5% BVL inclusion. The highest levels of dietary BVL were associated with lower weight gain; however, the production costs were reduced by BVL inclusion, and return per kilogram BW gain was improved. In light of our findings, BVL seems to be a feedstuff that can be used for sustainable lamb diets (up to 7.5% DM of diet) as it is associated with favorable feeding costs without deleterious effects on growing performance. For greater scientific reach, further studies should consider the use of BVL focusing on rumen function and quality of products.

## Data Availability

All of the required data have been presented in our article.

## Abbreviations:

ADF: acid detergent fiber
ADG: average daily gain
ADL: acid detergent lignin
AH: alfalfa hay
AIA: acid insoluble ash
BVL: *Berberis vulgaris* leaf
CCW: cold carcass weight
CP: crude protein
DM: dry matter
DMI: dry matter intake
EE: ether extract
FCR: feed conversion ratio
FE: feed efficiency
GP: gas production
HCW: hot carcass weight
ME: metabolizable energy
NDF: neutral detergent fiber
NFC: nonfibrous carbohydrates
OM: organic matter
PC: phenolic compounds
PSMs: plant secondary metabolites
SCFA: short chain fatty acid concentrations
TMR: total mixed ration
TT: total tannin
